# Influences of preoperative metformin on immunological factors in early breast cancer

**DOI:** 10.1007/s00280-020-04092-2

**Published:** 2020-06-12

**Authors:** Takahiro Tsukioki, Tadahiko Shien, Takehiro Tanaka, Yoko Suzuki, Yukiko Kajihara, Minami Hatono, Kengo Kawada, Mariko Kochi, Takayuki Iwamoto, Hirokuni Ikeda, Naruto Taira, Hiroyoshi Doihara, Shinichi Toyooka

**Affiliations:** 1 Department of General Thoracic Surgery and Breast and Endocrine Surgery, Okayama University Graduate School of Medicine, Dentistry, and Pharmaceutical Sciences, 2-5-1 Shikata-cho, Kita-ku, Okayama, 7008558 Japan; 2 Department of Pathology, Okayama University Hospital, 2-5-1 Shikata-cho, Kita-ku, Okayama, 700-8558 Japan

**Keywords:** Breast cancer, Metformin, Preoperative, Tils, CD8, PD-L1

## Abstract

**Purpose:**

Metformin has been suggested to possibly reduce cancer risk. However, the mechanism underlying the positive effects of metformin on cancer treatment remains unclear. We conducted a prospective study to evaluate the effects of preoperative metformin in patients with early breast cancer.

**Method:**

We evaluated the effects on immunological factors (TILs, CD4 + , CD8 + , PD-L1, IFNγ and IL-2) by comparing core needle biopsies (CNB) obtained before metformin treatment with surgical specimens. Seventeen patients were enrolled in this prospective study from January to December 2016. We also analyzed 59 patients undergoing surgery during the same period to reveal the correlation of immune factors between CNB and surgical specimen.

**Result:**

There was a moderate correlation between CNB and surgical specimens on TILs and CD8 + lymphocyte. (TILs Rs = 0.63, CD4 + Rs = 0.224, CD8 + Rs = 0.42) In the metformin group, TILs increases were confirmed in five (29%) patients, while a decrease was confirmed in two (12%). The expressions of CD4 + and CD8 + by TILs were increased in 41% and 18% of surgical specimens, respectively. However, TILs number (*p* = 0.0554), CD4+ (*p* = 0.0613) and CD8 + (*p* = 0.0646) expressions did not significantly increased. Furthermore, IFNγ expression appeared to be increased in response to metformin (*p* = 0.08).

**Conclusion:**

Preoperative metformin tends to increase TILs, as well as the numbers of CD4 and CD8 positive lymphocytes, and IFNγ levels. Metformin might improve immune function and have a possibility of chemo-sensitivity and thereby increase the effectiveness of immunotherapy, based on the results of this preliminary study.

**Electronic supplementary material:**

The online version of this article (10.1007/s00280-020-04092-2) contains supplementary material, which is available to authorized users.

## Introduction

Breast cancer is among the major causes of cancer-related death in women worldwide. While significant progress has been made in the diagnosis and treatment of breast cancer, the clinical outcomes of patients are still discouraging [1, 2]. Diabetes mellitus (DM) has an adverse impact on cancer. A systematic review indicated DM patients to have a 17% increased risk for breast cancer mortality [3]. One report has suggested that treating DM with metformin, that is one of the commonly prescribed drugs for type 2 DM, reduces the risk of breast cancer death as compared with sulfonylureas and insulin formulations [4]. However, the mechanism underlying the favorable effect of metformin on cancer epidemiology has yet to be investigated in detail.

Preclinical research using both breast cancer cell lines and mouse models subsequently showed that metformin represses cancer cell and xenograft growth. Some of the reported mechanisms are that metformin preferentially kills cancer stem cells through activation of AMP-activated protein kinase, leading to activation of FOXP 3, and the inhibition of m-TOR, a crucial signaling pathway for cellular proliferation and the survival of cancer cells, and also exerts an anti-tumor effect by enhancing immune function [5–7]. Of these mechanisms, the metformin’s effect on tumor microenvironment including immune functions is drawing attentions. Eikawa et al. showed that an anti-tumor effect of metformin is dependent on CD8+ T-cells in a murine model [7]. Nicole et al. reported that although tumor hypoxia inhibits T-cell function, metformin improves an inhibitor of tumor oxygen consumption and increases activation of T-cell [8].

Moreover, it is certain that the interplay between immune cells and tumor cells exerts a major influence on both the development and the progression of breast cancer [9]. Especially, the role of the cytotoxic T lymphocytes in tumorigenesis, has been explored extensively. T-cell infiltration in invasive breast cancer has been reported that secrete several inflammatory cytokines, for example interferon-γ (IFNγ), transforming growth factor beta (TGF-β), tumor necrosis factor alpha (TNFα) and interleukin-2 (IL-2). These cytokines then interact with other cytotoxic T-cells and upregulate the MHC class I and II molecules, as well as other antigen display co-factors in neoplastic cells [10]. We believe this process is an essential part of immune-mediated anti-tumorigenic effects.

The immune factor has an important role in clinical practice. Tumor infiltrating lymphocytes (TILs) are known to be prognostic factors as well as markers predicting chemo-sensitivity [11, 12]. Mao et al. conducted a meta-analysis which showed that higher levels of TILs correlated with the pCR rate in response to neoadjuvant chemotherapy (NAC) [15]. Other reports show that TILs is a significant predictor of the pathologic complete response in patients with triple-negative (TN) and human epidermal growth factor receptor-2 (HER2) positive breast cancer subtypes [13, 14]. In addition, various TIL subsets have different roles in breast cancer progression. CD4 + and CD8 + lymphocytes play crucial parts in determining cancer treatment and high intra-tumoral lymphocyte counts were found to be associated with better survival in breast cancer patients [16]. And higher levels of CD8 + T-lymphocytes in pre-treatment biopsy specimens predicted a better pathological response to NAC [15]. And cancer immunotherapy (PD-1, PDL-1 antigen) approaches have become among the most effective treatments for metastatic breast cancer [17].

Thus, we speculate that metformin improves immune function and favorably modifies the tumor environment. To our knowledge, there are no prior studies examining the influences of metformin on TILs, CD4 + , CD8 + , IFNγ, IL-2 and PDL-1 in patients without DM. We conducted this prospective study to confirm the effects of preoperative metformin in patients with early breast cancer (metformin study).

## Materials and methods

To evaluate the effects of metformin, we decided to do two studies. First, we gathered patients who had been diagnosed with breast cancer by CNB and after that they took metformin in the preoperative period, and we compare CNB that was pre-metformin with surgical specimen that was post-metformin. (metformin study) Second, because of breast cancer is known to have tumor heterogeneities, it is not uncertain to compare CNB with surgical specimen on immune functions. So, we should analyze the correlations of immune functions between CNB and surgical specimens. We assessed patients who underwent surgery and did not take metformin during the same period. (correlation study).

The patients, who had newly diagnosed stage I or IIA breast cancer and no history or evidence of DM based on blood biochemistry, were not taking metformin before enrollment in this study. All participants were enrolled between January and December 2016 at Okayama University Hospital. All participants were then administered a daily dose of metformin orally for 2 weeks before surgery. All 17 patients had the same oral metformin dosing schedule: 500 mg daily for 3 days (250 mg twice after the morning and evening meal), increased to 750 mg daily for next 4 days (250 mg three times after every meals), and if no adverse effects were observed, the dose was increased to 1000 mg daily for next 7 days before surgery (500 mg twice after the morning and evening meal).

Patients who received NAC or had recurrent tumors were excluded. Hematoxylin–eosin (HE)-stained slides of CNB and matched surgical specimens were reviewed retrospectively. We evaluated the expressions of and concordance between immuno-histological factors in CNB and surgical specimens from the same patient.

All clinical data were retrospectively extracted from our institution’s electronic medical records system. Metformin study was approved by the Ethics Committee of Okayama University Hospital (UMIN 000,014,090) and adhered to the Declaration of Helsinki. We obtained consent in writing from each patient. In addition, correlation study was approved by the Ethics Committee of Okayama University Hospital and adhered to the Declaration of Helsinki.

### Immunohistochemistry

Tumor morphology was evaluated using conventional HE-staining. Estrogen receptor (ER)/ progesterone receptor (PR) and HER2 were assessed according to standard guidelines. Nuclear staining ≧ 1% for estrogen receptors (ER) or progesterone receptors (PgR) was considered to be positive. HER2 positivity was defined as 3 + receptor overexpression on immunohistochemical (IHC) staining or gene amplification on fluorescence hybridization using a HER2/CEP ratio ≧ 2.0. The expressions of ER, PgR, HER2 and the Ki67 labelling index (Ki67) in both CNB and surgical specimens were determined.

Prior to IHC staining for immune factors, the tumor specimens were fixed in 10% formaldehyde solution and embedded in paraffin, then sliced into 4-μm-thick sections. We employed the Ventana Discovery XT automated platform (Ventana Medical Systems: Roche Tissue Diagnostics). Primary monoclonal antibodies directed against CD4 (SP35 Rabbit monoclonal antibody; Roche Tissue Diagnostics), CD8 (SP57 Rabbit monoclonal antibody; Roche Tissue Diagnostics), IFNγ (ab9657 Rabbit anti-interferon gamma antibody; Abcam), IL-2 (EPR2780 Rabbit monoclonal antibody; Abcam) and PD-L1 (22C3 Mouse monoclonal antihuman PD-L1 antibody; Dako North American) were used.

**Fig. 1:**
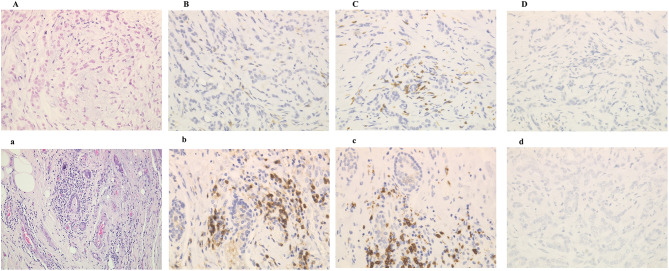
The expression of TILs, CD4 + , CD8 + and PD-L1 both CNB and surgical specimens among the same patient. There was increasing of TILs, CD4 + and CD8 + lymphocyte. TILs: (A, **a**), CD4 + : (B, **b**), CD8 + lymphocytes: (C, **c**), PD-L1: (D, **d**)

To determine the efficacy of metformin, we evaluated TILs, as well as CD4, CD8, IFNγ, IL-2 and PDL-1 expressions, in both CNB and surgical specimens (Fig. 1).

### Evaluation of TILs

HE-stained sections were utilized for evaluation for TILs. The percentages of stromal lymphocytes, serving as a predefined criterion in Denkert’s reports, were evaluated by one highly experienced observer [14, 18, 19]. Stromal TILs were measured as the percentage of immune cells in stromal tissue within the tumor that showed a mononuclear immunological infiltrate. Heterogeneous distributions were documented in nearly all sections. Therefore, hot spots, cold spots, and Tertiary Lymphoid Structure were not taken into consideration in conducting the measurements, with just one representative area being selected and evaluated. We divided the proportions of TILs into 10% increments. TILs were categorized according to three cut-off values, as a dichotomous variable (0 to 10%: negative, 11–30%: low, 31–50%: intermediate, over 51%: high). We also decided whether there was a 20% difference between the CNB and the surgical specimen.

### Evaluation of CD4 + and CD8 + T-lymphocytes, IFNγ, IL-2 and PD-L1

Based on previous studies, CD4 + and CD8 + expressions were calculated using the average of stained TILs in the area that was maximally stained, viewed at 400 × magnification [20, 21]. We randomly selected two different high-power fields and counted those expressing CD4 + or CD8 + in 100 lymphocytes.

The semi-quantitative H scoring system was employed, using whole tissue sections, to evaluate the presence of IFNγ and IL-2. The H score was calculated by multiplying the percentage of positive cells (tumor and immune) by a factor representing the intensity of immune-reactivity (1 for weak, 2 for moderate, and 3 for strong), giving a maximum score of 300. A score below 50 was considered negative and a score of over 50 was positive.

PD-L1 expression was categorized as positive when staining of the tumor-cell membrane (at any intensity) was observed at pre-specified expression levels of 1%, 5%, or 10% of cells in a section that included at least 100 tumor cells that could be evaluated [17]. As judging PD-L1 expression is difficult, we asked an experienced pathologist to determine the expression rates.

### Statistical analysis

This study was a non-planned, retrospective, exploratory project, for which all available cases were used without a predefined sample size calculation to detect a specific effect size or reach a certain level of power. Data were analyzed with EZR (version 1.37) [22]. *P* values < 0.05 were considered to indicate a statistically significant difference.

The immune related factors of CNB and surgical specimens were compared using Spearman’s paired rank correlation coefficient (Rs). To evaluate the correlation between the TILs, CD4 + and CD8 + lymphocytes in CNB and surgical specimens, Rs was calculated. Rs of < 0.4, 0.4–0.7, 0.7–0.9, and > 0.9 indicated poor, moderate, good, and excellent reliability, respectively.

To evaluate and compare the effects of preoperative metformin, we defined changes in TILs, CD4 + and CD8 + lymphocytes exceeding 20% as positive. The Related-Samples Wilcoxon Signed Rank test was performed for comparisons of the expressions of all immune factors.

## Results

### Correlation of TILs, CD4 + and CD8 + T-lymphocyte numbers between CNB and surgical specimens

Fifty-nine patients were evaluated as a correlation group. Patient characteristics are presented in Table 1. The median age was 65 (32–87) years. The median tumor size was 15 mm (range 2–52). Invasive ductal carcinoma accounted for 56 (95%) and invasive lobular carcinoma for 3 (5%) cases. Histological grade was III in 22 (37%), II in 7 (12%) and I in 30 (51%) cases. The Ki67 index was high (over 30%) in 22 (37%), intermediate (15–29%) in 17 (29%) and low (under 14%) in 20 (34%) cases. The breast cancer subtype was luminal A type (ER + , HER2 -, Ki67 < 15%) in 19 (32%), luminal B type (ER + , HER2-, Ki67 > 15%) in 23 (39%), luminal HER2 type in 4 (7%), HER2-enriched type in 4 (7%) and TN type in 9 (15%) cases (Table 1). The status of common biological markers did not differ between CNB and surgical specimens (Supplementary Table 1).

**Table 1:** Clinicopathological characteristics of the correlation study group (*n* = 59)

	Values
Age	
Median (range)	65 (32–87)
Distribution—no. (%)	
> 50	13 (22)
≤ 50	46 (78)
Tumor size—median (range: mm)	15 (2–52)
T1a—no. (%)	3 (5)
T1b	15 (25)
T1c	22 (37)
T2	18 (31)
T3.T4	1 (2)
Tumor grade (surgical specimen)—no. (%)	
I	30 (51)
II	7 (12)
III	22 (37)
Ki67 index—no. (%)	
0–14	20 (34)
15–29	17 (29)
30	22 (37)
Lymph node metastasis—no. (%)	
Positive	12 (20)
Negative	47 (80)
Subtype (surgical specimen)—no. (%)	
Luminal A	19 (32)
Luminal B (HER2 negative)	23 (39)
Luminal B (HER2 positive)	4 (7)
HER2-enriched type	4 (7)
Triple negative	9 (15)

TILs in CNB were negative in 37 (63%), low in 18 (30%), intermediate in 4 (7%) and high in 0 cases. The corresponding values in surgical specimens were 34 (58%), 16 (27%), 4 (7%) and 5 (8%). The median numbers of lymphocytes expressing CD4 + and CD8 + were 34 and 27, respectively, in CNB and surgical specimens. We also evaluated 20 intervals of the surgical specimen scores, yielding CD4 + numbers of 17 (29%: 0 to 20), 24 (41%: 21 to 40) and 18 (30%: over 41) for CNB and CD8 + numbers of 19 (32%), 30 (51%) and 10 (17%), respectively, for surgical specimens. (Supplementary Table 1) We attributed this discordance to a difference in the numbers of lymphocytes exceeding 20% between the CNB and surgical specimens. The total concordance rates, no difference, of the levels of CD4 + and CD8 + lymphocytes were 25 (43%) and 29 (49%), respectively (Supplementary Table 2).

Correlation analysis showed moderate positive TILs correlations between CNB and resected specimens (Rs = 0.63, *p* = 0.0000000872). We evaluated tumor size, tumor grade, subtype and Ki67 index results. All subgroup analyses showed positive correlations. (data not shown) Most notably, tumor size, Grade III, HER2-enriched type and a high Ki67 index tended to show significant positive correlations (the Rs of these factors were all over 0.7). For TIL subsets, correlations with CD4 + were poor (Rs = 0.224, *p* = 0.0875), while moderate correlations with CD8 + were noted (Rs = 0.42, *p* =  0.00917) (Table 2).

**Table 2:** Correlations between CNB and surgical specimens, compared using the paired Spearman’s rank correlation coefficient (Rs), in the correlation study group

	Spearman’s Rs	*P* value
All patients	*r* = 0.63	*p* < 0.001
Tumor size		
T1 (*n* = 40)	*r* = 0.58	*p* < 0.001
T2,3,4 (*n* = 19)	*r* = 0.745	*p* < 0.001
Tumor Grade		
I (*n* = 30)	*r* = 0.469	*p* = 0.00898
II (*n* = 7)	*r* = 0.558	*p* = 0.193
III (*n* = 22)	*r* = 0.789	*p* < 0.001
Ki67 index		
< 30% (*n* = 37)	*r* = 0.431	*p* = 0.00772
≥ 30 (*n* = 22)	*r* = 0.765	*p* < 0.001
Subtype		
Luminal A	*r* = 0.424	*p* = 0.00465
Luminal B (HER2 negative)	*r* = 0.327	*p* = 0.16
Luminal B (HER2 positive)	*r* = 0.531	*p* = 0.0097
HER2-enriched type	*r* = 0.937	*p* < 0.001
Triple negative	*r* = 0.687	*p* = 0.00409
CD4 + lymphocytes	*r* = 0.224	*p* = 0.0875
CD8 + lymphocytes	*r* = 0.42	*p* = 0.00917

### Distributions of and changes in TILs, CD4 + and CD8 + lymphocytes in CNB and surgical specimens from patients receiving metformin preoperatively

Seventeen patients were enrolled in the prospective metformin study. Patient characteristics are presented in Table 3. The median age was 58 (36–74). The median tumor size was 13 mm (2–23). Histological grade was III in six (35%), II in six (35%) and I in five (30%) cases. Nine patients had lymphatic invasion, but none had evidence of vascular invasion. The Ki67 index was high (over 30) in 6 (35%), intermediate (15–29) in 6 (35%) and low (under 14) in 5 (30%) cases. The breast cancer subtype was luminal A type (ER + , HER2-, Ki67 < 15%) in six (35%), luminal B type (ER + , HER2-, Ki67 > 15%) in five (30%), luminal HER2 type in one (6%) and TN type in five (30%) cases. The CNB specimens obtained before metformin and the surgical specimens did not differ in terms of biological markers. Five patients (29%) were lymph node-positive. Nearly all characteristics and pathological biomarkers were differed minimally between the metformin and correlation groups (Table 3).

**Table 3:** Clinicopathological characteristics and tumor biology of the metformin study group (*n* = 17)

	Values
Age	
Median (range)	58 (36–74)
Distribution—no. (%)	
> 50	4 (24)
≤ 50	13 (76)
Tumor size—median (range: mm)	13 (2–23)
T1a—no. (%)	4 (24)
T1b	2 (12)
T1c	10 (59)
T2	1 (6)
T3.T4	0 (0)
Tumor Grade (surgical specimen)—no. (%)	
I	5 (30)
II	6 (35)
III	6 (35)
Ki67 index—no. (%)	
0–14	5 (30)
15–29	6 (35)
30	6 (35)
Lymph node metastasis—no. (%)	
Positive	5 (29)
Negative	12 (71)
Subtype (surgical specimen)—no. (%)	
Luminal A	6 (35)
Luminal B (HER2 negative)	5 (30)
Luminal B (HER2 positive)	1 (6)
HER2-enriched type	0 (0)
Triple negative	5 (30)

Fifteen patients were able to take metformin for 2 weeks, as planned. Two patients experienced nausea (GradeI) 3 days after starting metformin, and therefore discontinued this medication.

The distributions of TILs, CD4 + and CD8 + lymphocytes are shown in Table 4. The TIL expression results before taking metformin were negative in 15 (89%) and low in 2 (11%) patients, while after metformin 8 (47%) cases were negative, 8 (47%) had low and 1 (6%) showed intermediate expression. And CD4 + lymphocytes expression was that 5 (29%) patients were over 20%, while after metformin there were 12 (71%). CD8 + lymphocytes expression was that 6 (35%) were over 20%, while after metformin there were 10 (59%) (Table 4).

**Table 4:** Changes of immune-related factors expression before and after metformin administration

	Pre-metformin: CNB—no. (%)	Post-metformin: surgical—no. (%)
TILs		
Negative:0–10%	15 (89)	8 (47)
Low:11–30%	2 (11)	8 (47)
Intermediate:31–50%	0 (0)	1 (6)
High: > 50%	0 (0)	0 (0)
CD4 + lymphocytes		
0–20%	12 (70)	5 (29)
21–40%	3 (17)	7 (41)
41–60%	1 (6)	4 (24)
> 60%	1 (6)	1 (6)
CD8 + lymphocytes		
0–20%	11 (65)	7 (41)
21–40%	6 (35)	10 (59)
41–60%	0 (0)	0 (0)
> 60%	0 (0)	0 (0)
IFNγ positive: H score ≥ 50	2 (15)	6 (35)
IL-2 positive: H score ≥ 50	1 (6)	2 (12)
PD-L1 positive	4 (24)	7 (41)

We determined the percentages of TILs expressing CD4 + and CD8 + . We defined a change of at least 20% as indicating metformin efficacy. Comparison of the results obtained before versus after metformin showed an increase in seven (42%) cases, while one (6%) showed a decrease in TILs. 9 cases (53%) had increased CD4 + , while four (24%) showed a decrease. Six cases (35%) had increased CD8 + and one (6%) showed a decrease (Supplementary Table 3).

But there were no significant differences in the change of TILs, CD4 + and CD8 + lymphocytes, comparing the patients increasing more than 20% or not (Table 5).

**Table 5:** Statistical analysis of the preoperative metformin influence, compared using the Related-Samples Wilcoxon Signed Rank test

	Increasing—no. (%)	No increasing—no. (%)	*P* value
TILs	7 (42)	10 (58)	*p* = 0.0545
CD4 + lymphocytes	9 (53)	8 (48)	*p* = 0.0613
CD8 + lymphocytes	6 (35)	11 (65)	*p* = 0.0646
IFNγ	5 (38)	8 (62)	*p* = 0.0803
IL-2	6 (35)	11 (65)	*p* = 1
PD-L1	3 (18)	14 (82)	*p* = 0.572

### Distributions of and changes in IFNγ, IL-2 and PD-L1 in CNB and surgical specimens from patients receiving metformin preoperatively

We judged IFNγ and IL-2 expressions by the H score. IFNγ expression in CNB was positive in two cases (15%) preoperatively, while six (35%) cases were positive after metformin administration. IL-2 expression in CNB was positive in one case (6%) preoperatively, while two (12%) were positive after taking metformin.

PD-L1 expression was judged as being negative (0%), low (1–9%) or high (≥ 10%). Thirteen (76%) cases were negative, two (12%) had low and two (12%) had high PD-L1 expression in CNB. The corresponding values in surgical specimens were ten (59%), five (30%) and two (12%) (Table 4).

Comparisons of the CNB and surgical specimens yielded no statistically significant differences in the rises in the levels of IFNγ (*p* = 0.0803), IL-2 (*p* = 1) and PD-L1 (*p* = 0.572) (Table 5).

## Discussion

Interpreting limited samples, such as CNB, raises questions about both sampling adequacy and error. Regarding the points, the extent and potential effects of sample type and the spatial heterogeneity of tumor immune infiltrates have yet to be examined in sufficient detail. The clinical and scientific importance of assessing TILs in breast cancer has been highlighted by recent efforts to standardize the histologic interpretation of TILs in patient samples. However, neither TIL heterogeneity nor the adequacy of core biopsy samples versus tissue sections has been fully documented [23]. This difference might be attributable to the limited area of tumor tissue biopsied, with indefinite tumor borders, in the CNB. However, Cha et al. reported there was less than 5% differences between TILs of CNB and surgical specimen [24]. Compared with their study, there was slightly differences of TILs between CNB and surgical specimen in our study, but Rs showed moderately positive correlations. (Rs = 0.63) Thus, we concluded that TILs in CNB specimens constitute a reliable indicator of the TIL values for the entire surgically resected breast tumor. In addition, in clinical settings, sequential tumor core biopsies have become accepted in NAC and window-of-opportunity studies as a means of assessing early evidence of the therapeutic efficacy of an agent or a treatment strategy [25–27]. These have included neoadjuvant endocrine trials [28, 29] and the use of novel agents [27] in window-of-opportunity studies. These trials have identified the Ki67 index at 2 weeks as a predictor of relapse free survival [28] or efficacy [30] and as a prognostic marker for adjuvant chemotherapy [31, 32]. Therefore, it is important to determine whether CNB reflects the biology of the entire tumor.

The mechanism underlying the effects of metformin on the cancer remains uncertain. In chronic infectious diseases and cancer, CD8 + T cells specific for viral and/or tumor antigens undergo repeated TCR stimulation due to the presence of persistent pathogens or cancer cells and gradually lose their ability to secrete IL-2, TNFα, and IFNγ, eventually undergoing apoptotic elimination in a process known as immune exhaustion [33]. Eikawa revealed that metformin prevents apoptosis of CD8 + TILs and induces multifunctional CD8 + effector memory T-cells expressing the exhaustion marker Tim-3 in an in vivo murine study [7]. Furthermore, cancer immunotherapy (PD-1, PDL-1 antigen) has now been recognized as being among the most effective strategies for treating lung cancer and melanoma. In metastatic breast cancer, immunotherapy yields favorable outcomes. Immunotherapy efficacy may depend on TILs. If so, developing methods of increasing TILs may become an increasingly important research goal.

Since 1957, immuno-editing has been identified in a wide range of tumor progression forms. However, the relationships between tumors and factors comprising the immune system remain complex and still are not fully understood. Yan et al. reported that TILs with CD8 + are the main effector cells in the immune response, being associated with better disease-free survivals, but not improved overall survivals. CD4 + lymphocytes are composed of both T helper and regulatory cells, such that their roles are highly complex. Furthermore, according to Mao Y et al.’s meta-analysis, CD4 + lymphocytes are not prognostic markers for breast cancer [15]. However, CD4 + data are very limited, and more prospective studies are needed to confirm their prognostic value in breast cancer. According to these evidences, if metformin improves immune function and increases the number of TILs, CD4 + and CD8 + lymphocytes, preoperative metformin administration would be expected to enhance chemotherapy effects. We did not identify a statistically significant increase in TILs, CD4 + or CD8 + lymphocytes with preoperative metformin, but an increasing trend was noted.

These non-statistically significant results might be due to the metformin administration being short-term and low dose, to the small number of patients and to there being few large tumors and/or discordance among the breast cancer types. A previous randomized study examined the effects of metformin in non-DM patients. Ko et al. reported that patients taking 1000 mg metformin for 6 months had a significantly greater decline in glucose, body mass index and HbA1c levels [34]. In our study, we took metformin for only 2 weeks. It may not be effective on the human unless taking several months as this study. However, we worried that taking metformin for a longer time and delaying surgery might not be feasible in actual clinical settings, we decided the 2 weeks periods from diagnosis until surgery. We consider that the safety of taking 1000 mg metformin in non-DM patients has been proved, we think that we can increase the internal dose of metformin (1000 mg/day). In addition, there are not established evaluation methods on immune factors. In particular, H-score is not popular because the method of calculation is confusing.

If taking metformin has some influences on the immune system, it would be possible to improve or achieve a synergistic effect on systemic therapy like chemotherapy, hormone and any targeting therapies. And if the effects of metformin on immune function require longer treatment to manifest, we should be continued postoperatively or be combined with adjuvant therapies. Nicole et al. showed that combination of metformin with PD-1 blockade improved intra-tumoral T-cell function and tumor clearance. As a result of modifications of tumor microenvironment, there might be some possibility to release immunotherapy resistance [8]. At present, there are ongoing clinical trials including the following: “A Phase III Randomized Trial of Metformin vs Placebo in Early Stage Breast Cancer (NCT01101438)”, “A Study of Liposomal Doxorubicin + Docetaxel + Trastuzumab + Metformin in Operable and Locally Advanced HER2 Positive Breast Cancer (NCT02488564)”, “Neoadjuvant Toremifene With Melatonin or Metformin in Locally Advanced Breast Cancer”, and “Metformin Plus Neoadjuvant Chemotherapy in Breast Cancer”. Our study did not show a statistically significant difference, there were some tendency that metformin increases or has some effects on the immune functions.

## Conclusion

The TIL values in CNB specimens are a reliable indicator of the TIL status of the entire resected breast tumor.

Preoperative metformin tends to increase TILs, CD4 + , CD8 + lymphocytes and IFNγ, suggesting enhancement of immunological anti-tumor response in the patients with breast cancer.

## Electronic supplementary material

Below is the link to the electronic supplementary material.

Supplementary file1 (DOCX 16 kb)
