# Supplementary material for: Influences of preoperative metformin on immunological factors in early breast cancer
Source: Cancer Chemother Pharmacol. 2020 Jun 12;86(1):55–63. doi: 10.1007/s00280-020-04092-2 (PMC7338817; doi:10.1007/s00280-020-04092-2)
Supplement: Supplementary file 1 — Supplementary file1 (DOCX 16 kb) [file 280_2020_4092_MOESM1_ESM.docx]

Supplementary table 1 : The concordance of pathological and immune factors in the correlation study group.

|  | CNB specimen – no. (%) | Surgical specimen – no. (%) | Concordance rate of them |
| --- | --- | --- | --- |
| ER  positive  negative | 46(78)  13(22) | 46(78)  13(22) | 100% |
| PgR  positive  negative | 36(61)  23(39) | 45(76)  14(24) | 80% |
| HER2 score  0, 1+  2+  3+ | 47(80)  5(8)  7(12) | 47(80)  5(8)  7(12) | 100%  *All were FISH negative |
| TILs  negative :0-10%  low :11-30%  intermediate :31-50%  high :>50% | 37(63)  18(30)  4(7)  0(0) | 34(58)  16(27)  4(7)  5(8) | 88%  56%  25%  0% |
| CD4+ lymphocytes  0-20%  21-40%  41-60%  >60% | 12(20)  29(50)  12(20)  6(10) | 17(29)  24(41)  13(22)  5(8) | 18%  38%  15%  20% |
| CD8+ lymphocytes  0-20%  21-40%  41-60% | 21(36)  28(47)  10(17) | 19(32)  30(51)  10(17) | 53%  43%  20% |

Supplementary table 2 : Quantitative difference between CNB and surgical specimen in correlation study group.

| Difference | TILs – no. (%) | CD4+ lymphocytes – no. (%) | CD8+ lymphocytes – no. (%) |
| --- | --- | --- | --- |
| 0-19%  20-29%  30-39%  ≥40% | 49(83)  7(12)  1(2)  2(3) | 25(43)  13(22)  11(19)  10(16) | 29(49)  23(39)  6(11)  1(2) |

Supplementary table 3 : Quantitative changes of TILs, CD4+ and CD8+ lymphocyte expressions before versus after metformin.

|  | TILs – no. (%) | CD4+ lymphocytes – no.(%) | CD8+ lymphocytes – no.(%) |
| --- | --- | --- | --- |
| Increasing (≥20%) | 7(42) | 9(53) | 6(35) |
| 20%  30%  ≥40% | 4(24)  2(12)  1(6) | 3(18)  2(12)  4(24) | 3(18)  1(6)  2(12) |
| No change (0±19%) | 9(53) | 4(24) | 10(59) |
| Decreasing (≥20%) | 1(6) | 4(24) | 1(6) |
| 20%  30%  ≥40% | 0(0)  0(0)  1(6) | 3(18)  1(6)  0(0) | 1(6)  0(0)  0(0) |
